# Terahertz spectroscopy of anisotropic materials using beams with rotatable polarization

**DOI:** 10.1038/s41598-017-12568-0

**Published:** 2017-09-26

**Authors:** C. D. W. Mosley, M. Failla, D. Prabhakaran, J. Lloyd-Hughes

**Affiliations:** 1 Department of Physics, University of Warwick, Gibbet Hill Road, Coventry, CV4 7AL UK; 2 Department of Physics, Clarendon Laboratory, University of Oxford, Parks Road, Oxford, OX1 3PU UK

## Abstract

We introduce a polarization-resolved terahertz time-domain spectrometer with a broadband (0.3–2.5 THz), rotatable THz polarization state, and which exhibits minimal change in the electric field amplitude and polarization state upon rotation. This was achieved by rotating an interdigitated photoconductive emitter, and by detecting the orthogonal components of the generated THz pulse via electro-optic sampling. The high precision (<0.1°) and accuracy (<1.0°) of this approach is beneficial for the study of anisotropic materials without rotating the sample, which can be impractical, for instance for samples held in a cryostat. The versatility of this method was demonstrated by studying the anisotropic THz optical properties of uniaxial and biaxial oxide crystals. For uniaxial ZnO and LaAlO_3_, which have minimal THz absorption across the measurement bandwidth, the orientations of the eigenmodes of propagation were conveniently identified as the orientation angles that produced a transmitted THz pulse with zero ellipticity, and the birefringence was quantified. In CuO, a multiferroic with improper ferroelectricity, the anisotropic THz absorption created by an electromagnon was investigated, mapping its selection rule precisely. For this biaxial crystal, which has phonon and electromagnon absorption, the polarization eigenvectors exhibited chromatic dispersion, as a result of the monoclinic crystal structure and the frequency-dependent complex refractive index.

## Introduction

Recent advances in the generation and detection of terahertz (THz) radiation have enabled a wide range of intriguing material properties in the far-infrared region of the electromagnetic spectrum to be investigated. In particular, terahertz time-domain spectroscopy (THz-TDS) has matured into a powerful tool for characterizing the optical properties of materials at THz frequencies^1–3^. Many materials demonstrate anisotropic behavior at THz frequencies, such as birefringence created by anisotropy in the vibrational or electronic response^4,5^, and electro- and magneto-optical effects^6–8^. Designs of optical components for polarization control in the THz region, such as wire-grid polarizers (WGPs)^9^ and wave plates^10^, depend critically on the optical anisotropy. Therefore the accurate determination of the optical properties of anisotropic materials is important for both optical component design and fundamental physical research^11^.

The optical properties of a non-magnetic anisotropic crystal can be described by the dielectric tensor, ***ε***, as the electric displacement ***D*** = ***ε*** *ε*_0_ ***E*** is no longer necessarily parallel to the THz electric field ***E***. For light with angular frequency *ω* propagating in an anisotropic crystal with an arbitrary wavevector ***k*** there are two orthogonal normal modes, with polarization eigenvectors ***u***_1,2_. These normal modes each have different refractive indices. If the incident light is linearly polarized with components of ***E*** along ***u***_1_ and ***u***_2_, the transmitted light will in general be elliptically polarized, i.e. the medium acts as a wave plate. The polarization state of an electromagnetic wave is characterized by its ellipticity angle *χ*(*ω*), where *χ* = 0 for linear polarization and *χ* = ±45° for circular polarization, and its orientation angle *ψ*(*ω*)^11^.

One method by which the optical properties of anisotropic media can be studied is to rotate the sample^4,12^, changing the directions of ***u***_1,2_ relative to ***E***. While simple to perform at room temperature, at cryogenic temperatures or in high external magnetic fields rotating the sample is challenging. Also, if the sample is not perfectly aligned with the axis of rotation, different numbers of grains or domains in the sample may be probed at different angles. This is an important consideration in inhomogeneous materials such as lanthanum aluminate (LaAlO_3_), where the transmitted THz polarization state depends strongly on the size and number of domains probed^5^.

Alternatively to rotating the sample, the THz polarization state may be rotated by: changing the orientation angle of the generated beam during the emission process; rotating it using a half-waveplate; projecting it using a WGP. The latter two methods are used in spectroscopic ellipsometry, with WGPs favored for THz ellipsometry^13^. Ideally, a polarization rotation system should satisfy the following criteria: a minimal insertion loss for any components used, minimal change in ***E*** with rotation angle in terms of the amplitude |***E***| and polarization state (*ψ* and *χ*), precise and accurate determination of *ψ* and *χ*, and uniform operation over a wide bandwidth. Half-waveplates made of a single birefrigent material^5^ and metasurface-based polarization rotators^14^ are intrinsically narrowband and have finite loss, and are thus not ideal for broadband THz spectroscopy with arbitrarily rotatable THz pulses. Recently however, efforts have been made to produce metamaterial-based devices that operate over a broader frequency range^15–17^. Internal reflection within a prism offers broadband polarization rotation: for instance a half-waveplate with retardance close to *π* and varying by ±6° with frequency was reported^18^. These components are large and require collimated THz beams. A rotation of the generated THz beam has been shown for plasma-based THz emission^19^, which is reliant upon high pulse energy laser amplifiers with lower repetition rates. In terms of the more widely-used THz-TDS systems based on laser oscillators, photoconductive emitters are the THz source of choice for spectroscopy and imaging applications in custom-made and commercial systems. The coarse rotation (to 0°, 45° and 90°) of a wide-area photoconductive emitter has been reported, to alternate between vertical and horizontal THz emission^20^, with an accuracy in the orientation angle of 5°, a large ellipticity, and a variation in |***E***| by 40% on rotating the emitter by 90°. This summary of polarization-rotation methods is by no means exhaustive, however no scheme meets all the above criteria.

Here we introduce the method of rotatable polarization terahertz time-domain spectroscopy (RP-THz-TDS), which provides a convenient and powerful probe of the behavior of anisotropic materials at THz frequencies, based on rotating an interdigitated photoconductive emitter. We obtained *E*_*x*_ and *E*_*y*_ directly via electro-optic sampling, resolving the full THz polarization state (*ψ*, *χ*), without requiring the extra components, assumptions and data analysis required by ellipsometric methods. We show that this approach allows broadband polarization rotation, with linearly polarized THz pulses that can be rotated to arbitrary angles (accuracy <1.0°, precision <0.1°), and exhibiting a small ellipticity (accuracy <0.75°, precision <0.1°), across a frequency range of 0.3–2.5 THz. The method does not rely upon THz waveplates, and is therefore broadband, and does not require wire-grid polarizers, circumventing the problem of their poor extinction ratio. We demonstrate the applicability of this technique by investigating: (i) the finite ellipticity created by a wire-grid polarizer; (ii) the anisotropic behavior of two uniaxial materials, zinc oxide (ZnO)^21^ and lanthanum aluminate (LaAlO_3_)^5^; and (iii) anisotropy in the refractive index and absorption for the biaxial compound cupric oxide (CuO)^7,22^. Rotating the THz polarization state not only allows convenient access to the anisotropic optical properties at cryogenic temperatures or in high magnetic fields, but also ensures that the same area on the sample is probed at each angle, solving both of these issues related to rotating the sample.

## Results

### Rotatable polarization THz-TDS

**Figure 1:**
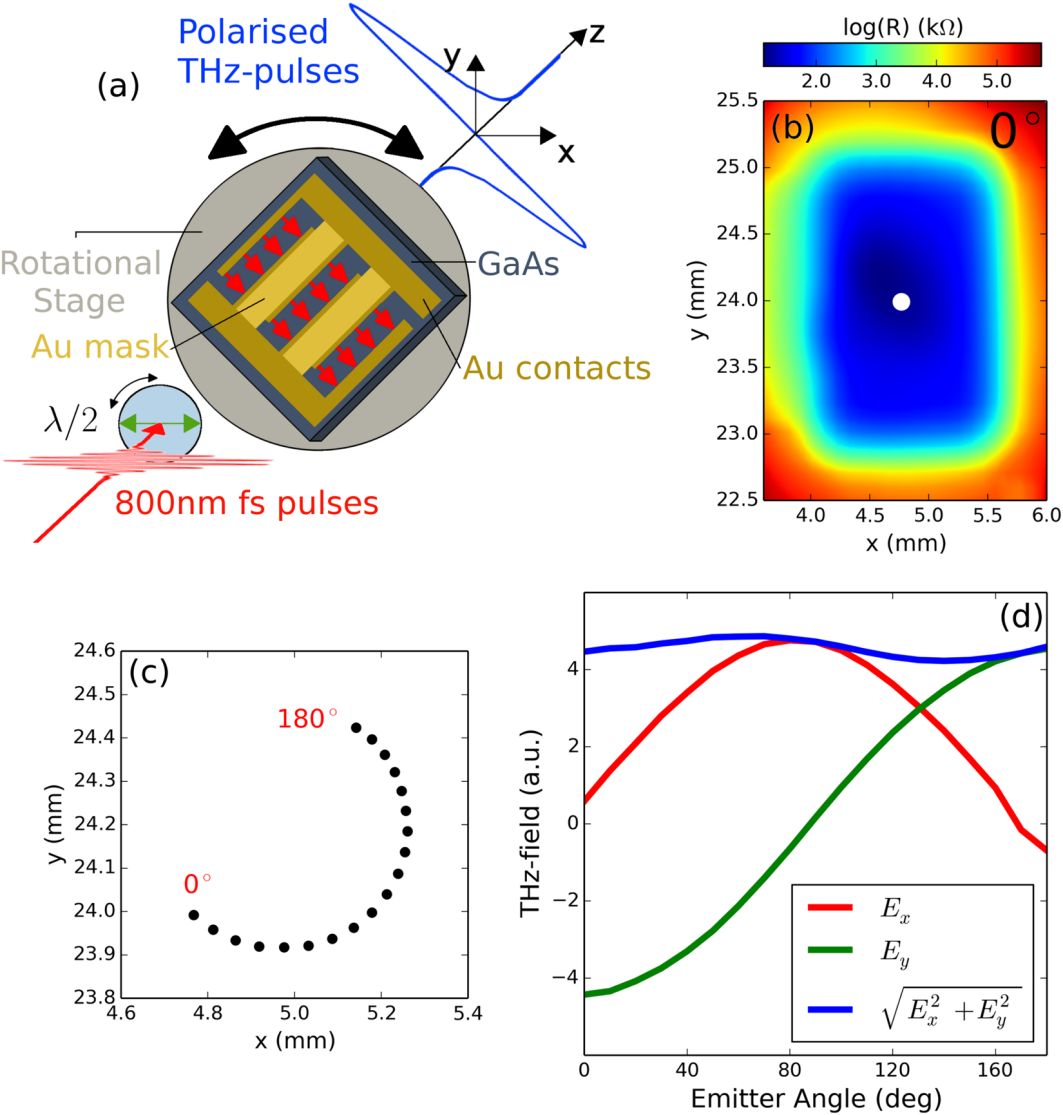
(**a**) Schematic (not to scale) of the interdigitated THz emitter mounted on a motorised rotation stage. The THz pulse (blue waveform) has a linear polarization parallel to the direction of the applied electric field (red arrows). (**b**) Map of the emitter’s resistance as it is raster scanned in the *x* − *y* plane, at *ψ*_em_ = 0° and under illumination. The device’s center is shown by the white dot. (**c**) The device’s center as a function of *ψ*_em_ (see text). (**d**) Dependence on *ψ*_em_ of the time-domain peak amplitude of the horizontal component (red curve), vertical component (green) and total amplitude (blue, ${({E}_{x}^{2}+{E}_{y}^{2})}^{\mathrm{1/2}}$) of the THz electric field, which is vertically polarized close to *ψ*_em_ = 0° and 180°, while it is horizontal close to *ψ*_em_ = 90°.

Figure 1(a) shows our scheme for THz-TDS with a rotatable, linear THz polarization state, which exploited an interdigitated photoconductive emitter mounted in a motorized rotation stage. Details of the emitter are given in the Methods section. Photoexcited carriers were accelerated perpendicular to the gold strips (as shown by the red arrows), defining the linear polarization state of the THz pulse. The orientation angle was then varied by rotating the rotation stage (black arrow).

On rotating the emitter it was apparent that its centre was close to, but not exactly on, the axis of the rotation stage. Thus under rotation the emitter was no longer centered on the THz generation beam, altering the radiated THz power. To overcome this problem we mounted the rotation stage on a motorized *xy*-stage and mapped the resistance of the device, under illumination, at different emitter angles *ψ*_em_ from *ψ*_em_ = 0° to *ψ*_em_ = 180° (limited by the emitter’s wiring). Such a map at *ψ*_em_ = 0° is shown in Fig. 1(b) together with the center of the device obtained with a ‘centre of mass’ calculation (white dot). The co-ordinates of the emitter’s center are shown in Fig. 1(c) for all values of *ψ*_em_: the emitter was off-centered from the stage’s rotation axis by about 0.3 mm. This calibration step allowed us to re-centre the emitter at each *ψ*_em_. A larger emitter area or more precise mounting on the rotation stage’s axis could avoid this *x* − *y* calibration step.

Polarization-resolved THz detection has been reported using photoconductive antennae with three and four contacts^20,23,24^, or by rotating a (110)-oriented electro-optic crystal^25^ or the polarization of the detection beam^26^ in electro-optic sampling. Here, the polarization state of the emitted THz radiation was evaluated by measuring its orthogonal components with the method proposed by van der Valk *et al*.^26^. In this variant of electro-optic sampling the gate beam propagates through a quarter-wave plate (QWP), a [111]-oriented ZnTe, a half-wave plate (HWP) and a Wollaston prism (WP)^26^. By considering the THz electric field’s components along the $\mathrm{[0}\overline{1}\mathrm{1]}$ and $[\overline{2}\mathrm{11]}$ axes of the [111]-ZnTe as ${E}_{0\overline{1}1}$ and ${E}_{\overline{2}11}$, respectively, the electro-optic signal Δ*I* can be written^26^:

1 $${\rm{\Delta }}I\propto [{E}_{\overline{2}11}\,\sin \,\mathrm{(2}\theta -4\delta )+{E}_{0\overline{1}1}\,\cos \,\mathrm{(2}\theta -4\delta )],$$

where *θ* is the angle between the WP and $\mathrm{[0}\overline{1}\mathrm{1]}$, and *δ* is the angle between the HWP and the WP.

To align the detection system we first found the HWP angle that minimized the vertical component of the gate beam after the WP, thus defining *δ* = 0. We then set the emitter at *ψ*_em_ = 0° in order to obtain vertically polarized THz radiation (i.e. along the *y*-axis), and rotated the ZnTe about its surface normal to find the maximum electro-optic signal, where *θ* = 0. Under these conditions, the electro-optic signal is ${\rm{\Delta }}I\propto {E}_{0\overline{1}1}$, and corresponds to the *y* component of the THz electric field, *E*_*y*_. The *x* component, *E*_*x*_, was then obtained by setting *δ* = 22.5° (see Eq. (1)) by precisely rotating the HWP, mounted in a motorized rotation stage.

The polarization state of an electromagnetic wave can be characterized by its ellipticity angle *χ*(*ω*), where *χ* = 0 for linear polarization and *χ* = ±45° for circular polarization^11^, and its orientation angle *ψ*(*ω*), which is the angle with respect to the *x*-axis, as defined by the [$\bar{2}11$]-axis of the ZnTe in our setup. We obtained *χ* and *ψ* by converting the complex THz spectra ${\tilde{E}}_{x}(\omega )$ and ${\tilde{E}}_{y}(\omega )$ into a circular basis using ${\tilde{E}}_{\pm }=|{\tilde{E}}_{\pm }|{e}^{i{\phi }_{\pm }}=({\tilde{E}}_{x}+i{\tilde{E}}_{y})/\sqrt{2}$, and by then using $\tan \,\chi =(|{\tilde{E}}_{-}|-|{\tilde{E}}_{+}|)/(|{\tilde{E}}_{+}|+|{\tilde{E}}_{-}|)$ and *ψ* = (*ϕ*_+_ − *ϕ*_−_)/2.

### Performance of rotatable polarization setup

Ideally, the RP-THz-TDS should have a minimal variation in the amplitude of the electric field $|{\boldsymbol{E}}|={({E}_{x}^{2}+{E}_{y}^{2})}^{\mathrm{1/2}}$ with emitter angle *ψ*_em_, in order to obtain a good signal-to-noise ratio for all polarization directions. The vertical and horizontal components of the peak THz electric field are shown in Fig. 1(d) versus *ψ*_em_ by the red and green lines, respectively. When the applied electric field in the emitter was vertical, close to *ψ*_em_ = 0° and 180°, the amplitude of *E*_*y*_ was a maximum, while *E*_*x*_ was greatest near *ψ*_em_ = 90°. A small offset in the emitter angle compared to the orientation angle can be identified, for instance by the small *E*_*x*_ component at *ψ*_em_ = 0. The total amplitude of the THz electric field |***E***| initially varied by 20% across the full range of rotation. A motorized HWP was then added to the THz generation beam, to co-rotate the pump polarization along with the emitter, keeping the pump’s polarization parallel to the gold contacts. This optional step reduced the variation in |***E***(*ψ*_em_)| to less than 7%, as shown by the blue curve in Fig. 1(d), ensuring that a good signal-to-noise can be obtained for all incident polarization states.

**Figure 2:**
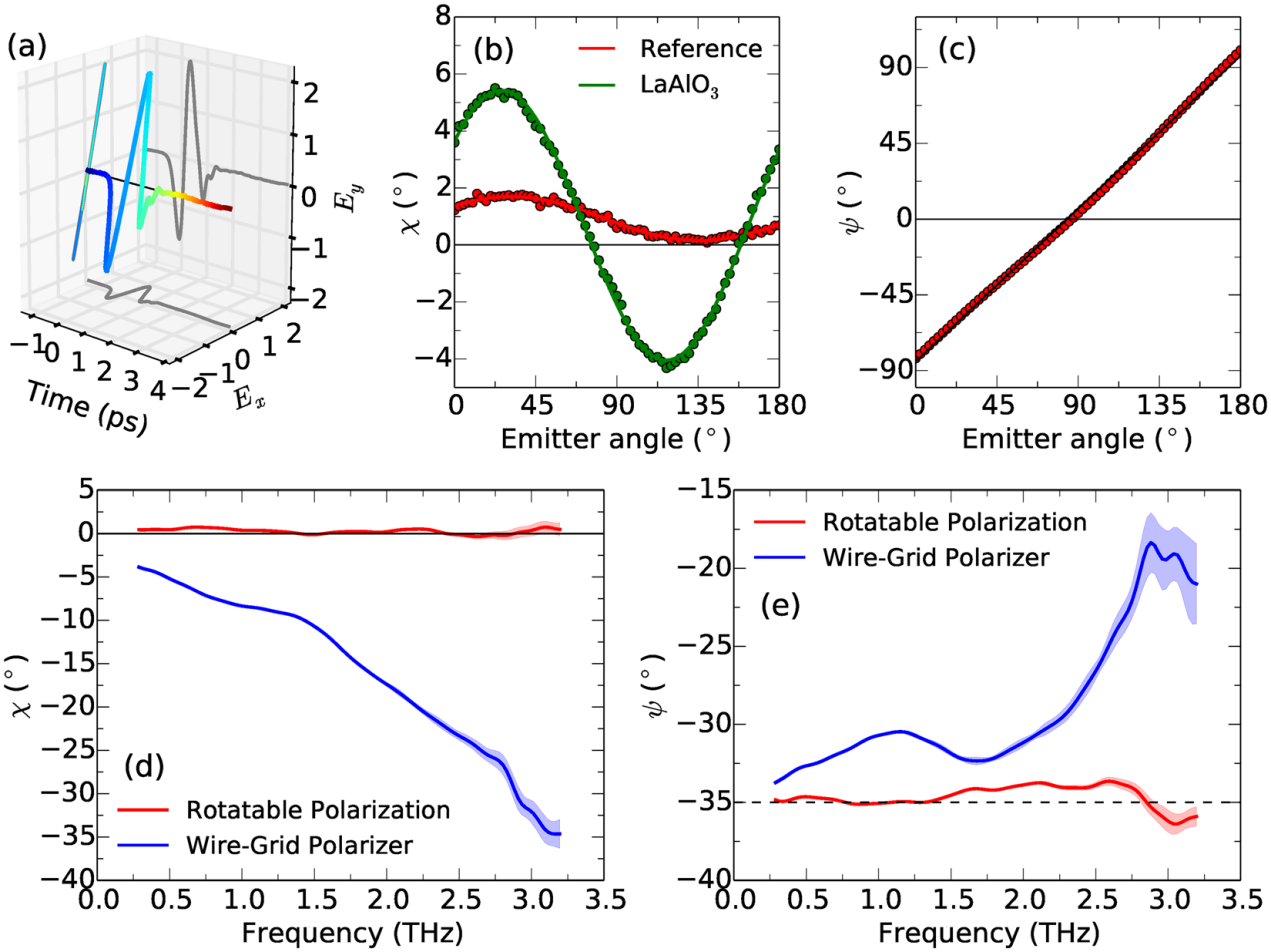
(**a**) Polarization-resolved time-domain waveform of an incident THz pulse polarized at *ψ*_em_ = 140°. (**b**) Experimental data (dots) and fits (lines) of the ellipticity of THz pulses incident and after transmission through LaAlO_3_. (**c**) Comparison of *ψ* and *ψ*_em_ of THz pulses. (**d**) and (**e**) Show the ellipticity and orientation angle, respectively, of THz pulses after transmission through a WGP acting on the incident pulse at an angle of 45° (blue lines), and without transmission through the WGP after the incident polarization state has been rotated 45° by rotating the emitter (red lines). Shaded regions show the standard deviation after 20 repeated scans. The dashed line in (**e**) represents the direction perpendicular to the wires of the WGP.

Further, an ideal RP-THz-TDS setup would exhibit minimal variation in ellipticity *χ* with emitter angle *ψ*_em_, and precise and accurate knowledge of *χ* and *ψ*. Polarization-resolved time-domain waveforms of the pulses were recorded at each *ψ*_em_ in 2.5° steps over a 180° rotation. One such time-domain waveform can be seen in Fig. 2(a), at *ψ*_em_ = 140°. The ellipticity at 1.0 THz as a function of *ψ*_em_ is reported in Fig. 2(b). The average *χ* of THz pulses from the emitter was 0.925°, which varied by 0.75° over a 180° rotation, demonstrating that the polarization remained close to linear for all *ψ*_em_. The small measured ellipticity may be an artifact of a slight misalignment of the gate and THz beams while propagating through the detection crystal^27^. However, this small ellipticity, reported here for the first time for an interdigitated THz emitter, is much smaller than the ellipticity $\chi \sim {10}^{\circ }$ of wide-area emitters^20^ and dipole antenna^28^. While a small quadrupole moment may contribute to the finite ellipticity in these two cases^28^, the small ellipticity in our case may result from the gold fingers, which in essence act as a wire-grid polarizer within the near-field of the generated THz radiation.

In order to determine precisely which emitter angle *ψ*_em_ corresponded to a horizontally polarized pulse (where *ψ* = 0°) the orientation angle of the incident pulse at 1.0 THz was measured as a function of emitter angle, shown in Fig. 2(c). A linear fit to the data gives an emitter angle of 83.85° for *ψ* = 0°. Thus the THz orientation angle *ψ*_in_ incident on a sample was calibrated precisely for each *ψ*_em_. Zero *ψ*_in_ corresponds to a horizontally polarized incident pulse and *ψ*_in_ = ±90° corresponds to vertically polarized incident pulses of opposite polarity.

The frequency response of the RP-THz-TDS system is reported in the following section, along with a comparison to an alternative polarization rotation method using a WGP, as often used in THz ellipsometry^13^ and polarimetry schemes^29^.

### Performance of RP-THz-TDS compared to WGP projection

Static or continuously rotatable WGPs are commonly used in polarization-resolved detection schemes to determine the orthogonal components of the electric field^29,30^. By projecting linear polarization states along different directions the direct detection of orthogonal components of ***E*** is not required. However, these methods suffer from the poor extinction ratio of WGPs^4,31^, which is frequency dependent. Further, there is a *π*/2 phase shift between light parallel to and perpendicular to the wires^32^ that introduces a finite ellipticity to the transmitted beam, which we show below.

To illustrate the advantages of the RP-THz-TDS method over polarization rotation via WGPs, we compared the polarization state produced using a WGP with that directly from our rotatable polarization setup. A perfect WGP at an arbitrary angle should fully transmit the component of the electric field perpendicular to the wires and transmit none of the component parallel to the wires, hence transmitting a polarization state rotated with respect to the original with a reduced amplitude and minimal ellipticity. A WGP was produced by UV photolithography, and consisted of gold wires with 8 *μ*m width and 20 *μ*m period on a semi-insulating GaAs substrate. The WGP was held in a manual rotation mount at the sample position, and was rotated to an arbitrary angle. This angle was precisely identified as *ψ*_in_ = −35.0°, by scanning *ψ*_in_ over a range of 20° in 0.625° steps around the approximate direction perpendicular to the wires and determining the angle at which the transmitted THz amplitude |***E***| was a maximum. The emitter was then rotated by 45° such that the THz pulse incident on the WGP would have equal components parallel and perpendicular to the wires. The frequency dependent ellipticity and orientation angle are shown by the blue lines in Fig. 2(d) and (e), respectively. The WGP produces a highly frequency-dependent elliptical pulse, which is approaching circular polarization for frequencies above 3.0 THz. This ellipticity occurs due to the finite transmission of the component of the THz electric field parallel to the wires; this component is the first time derivative of the incident field^32^, and is therefore phase-shifted by a factor of *π*/2 with respect to the orthogonal component perpendicular to the wires. The orientation angle also deviates significantly from the intended angle of *ψ* = −35.0°, by 2.0° in the best case (low frequency), and with larger change as the transmitted pulse becomes more elliptical.

The WGP was then removed and the emitter rotated back by 45°, to the same angle as defined by the WGP. The frequency dependence of the ellipticity and orientation angle is shown by the red lines in Fig. 2(d) and (e), respectively. The rotatable polarization method produces a linear THz pulse over the whole experimental bandwidth when rotated to an arbitrary angle, with an ellipticity of ≤0.75° and a statistical error (the precision) in the ellipticity of <0.05° over the 0.3–1.5 THz range (<0.1° from 0.3–2.5 THz) after 20 repeated measurements. The accuracy of the orientation angle was defined as the variation of *ψ*(*ω*) away from the mean^30^, and was <1.0° between 0.3–2.5 THz. The statistical error in the orientation angle was <0.05° between 0.3–1.5 THz (<0.1° from 0.3–2.5 THz).

The above demonstrates the drawbacks in using WGPs in polarization-resolved detection or in ellipsometric schemes to study anisotropic materials. While the above WGP may not be competitive with the best commercial WGPs (which have smaller periods), any WGP will introduce uncertainty into the identification of optical properties. Comparatively, RP-THz-TDS does not suffer from this issue, and has an accuracy and precision comparable to the best ellipsometric methods^30^. In the following sections we utilize RP-THz-TDS to study birefringent media that are uniaxial (ZnO and LaAlO_3_) or biaxial (CuO).

### RP-THz-TDS of uniaxial ZnO and LaAlO_3_

To demonstrate how RP-THz-TDS can be used to investigate anisotropic media, we first studied a uniaxial crystal, ZnO. With a hexagonal structure (space group C6mc), ZnO consists of alternating hexagonal stacks of Zn^2+^ and O^2−^ ions along the *c*-axis, with each Zn^2+^ ion coordinated tetrahedrally with the O^2−^, and vice versa. By considering the crystal’s symmetry the number of independent components of ***ε*** can be reduced. The dielectric tensor for a uniaxial crystal can be expressed as

2 $${\boldsymbol{\varepsilon }}=(\begin{array}{ccc}{\varepsilon }_{xx} & 0 & 0\\ 0 & {\varepsilon }_{xx} & 0\\ 0 & 0 & {\varepsilon }_{zz}\end{array}),$$

where *ε*_*xx*_ and *ε*_*zz*_ are the components of the dielectric tensor along the mutually orthogonal principal axes of the material *x*, *y* and *z*, *ε*_*xx*_ = *ε*_*yy*_, and *z* is the optical axis. Since the dielectric tensor is diagonal, the polarization eigenmodes ***u***_1,2_ are along the principal axes. For light beams propagating along the optical axis (where ${\boldsymbol{k}}\times \hat{{\boldsymbol{z}}}=0$) the refractive index is independent of the polarization direction, and hence there is no birefringence. When light propagates along any other direction (${\boldsymbol{k}}\times \hat{{\boldsymbol{z}}}\ne 0$) the polarization eigenmodes will have different propagation speeds, and hence the material is birefringent.

Here measurements were performed on a 0.6 mm thick single crystal of ZnO, oriented with the *a*-axis as the surface normal and the *c*-axis in the plane. The birefringence in the THz range can be linked to the differences in phonon mode frequency and strength for directions parallel to and perpendicular to *c* ^21^, and also to any additional anisotropy in the electronic contribution to the dielectric function.

**Figure 3:**
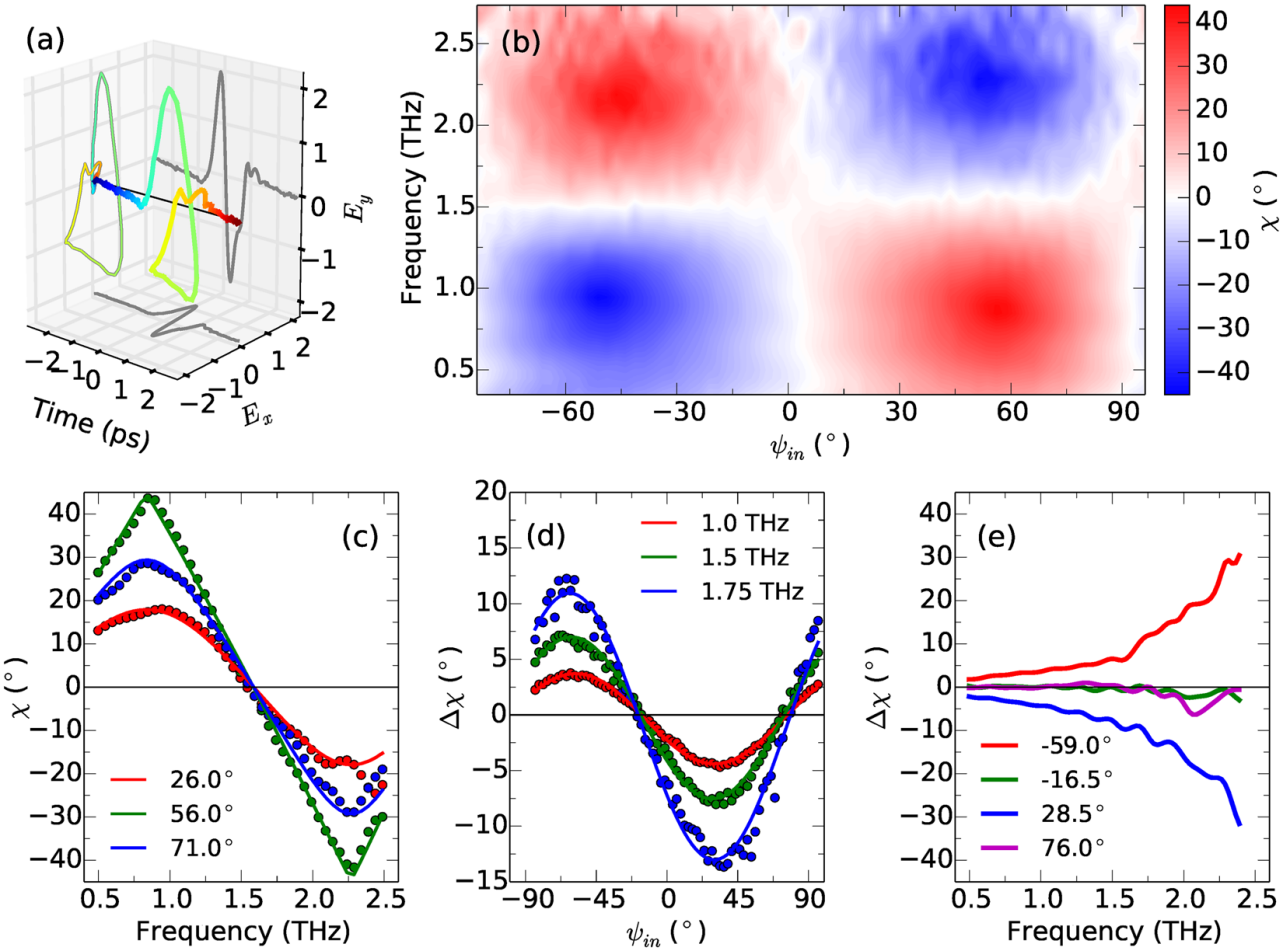
(**a**) Polarization-resolved time-domain waveform of a THz pulse, initially polarized at *ψ*_em_ = 140°, after transmission through ZnO. (**b**) Ellipticity of THz pulses after transmission through ZnO as a function of incident orientation angle. (**c**) Ellipticity as a function of frequency after transmission through ZnO, at three values of *ψ*_in_. Dots are experimental data and solid lines are calculated fits. (**d**) Experimental data (dots) and fits (lines) to the change in ellipticity of pulses after transmission through LaAlO_3_. (**e**) Change in ellipticity of THz pulses polarized along (*ψ*_in_= −16.5°, 76.0°) and midway between (−59.0°, 28.5°) the polarization eigenvectors of LaAlO_3_.

An example polarization-resolved time-domain waveform after transmission through ZnO is reported in Fig. 3(a), at the same emitter angle as Fig. 2(a), demonstrating how a phase delay between the components of the THz electric field propagating along the fast and slow axes of ZnO produces an elliptical polarization state. When *ψ*_in_ is midway between the fast and slow axes the ellipticity *χ* of the pulse transmitted through ZnO will be a maximum, while conversely *χ* = 0 when *ψ*_in_ is parallel to a polarization eigenvector. Therefore, by rotating the incident THz polarization state and measuring *χ* at each angle, the polarization eigenvectors can be accurately determined. Figure 3(b) shows the evolution of *χ* as a function of frequency after transmission through ZnO, as *ψ*_in_ is varied over 180°. Zero *χ* occurs around *ψ*_in_ = 4° and *ψ*_in_ = 94°, identifying the polarization eigenvectors. Tracing the evolution of *χ* with frequency, right- and left-handed circularly polarized states can be observed for certain *ψ*_in_ at 0.75 and 2.25 THz, where the ZnO is acting as a quarter-wave plate, and between these frequencies the polarization state becomes linear again at 1.5 THz, where the ZnO acts as a half-wave plate.

Figure 3(c) shows the frequency dependence of *χ* for *ψ*_in_ = 26.0° (red dots), *ψ*_in_ = 56.0° (green dots) and *ψ*_in_ = 71.0° (blue dots). The solid lines are the calculated ellipticity at each angle, represented by the same color, of an initially linearly polarized pulse transmitted through ZnO of the same thickness as the experimental sample. The birefringence Δ*n* = *n*_1_ − *n*_2_ of ZnO was assumed to be frequency dependent and was modeled as Δ*n* = Δ*n*_0_ + *αf*, where *f* is the frequency in THz. A quadratic component to the birefringence was also considered, but was found to be negligible in the experimental frequency range. Using this model and fitting to the experimental data gave Δ*n*_0_ = 0.14 and *α* = 0.011 THz^−1^. A linear fit to Δ*n* over the experimental frequency range 0.5–2.5 THz is reasonable, as the lowest infrared active phonon modes in ZnO occur at 11.5 and 12.5 THz for the *A*_1_ and *E*_1_ modes respectively^21^.

To further utilize our RP-THz-TDS setup we examined an inhomogeneous uniaxial crystal, LaAlO_3_. At room temperature LaAlO_3_ is a rhombohedral perovskite with space group $R\bar{3}c$ ^33^. The optical axis is in the [111] pseudocubic direction^5^. While ZnO forms a homogeneous single crystal, LaAlO_3_ is inhomogeneous with twin domains bounded along the [010] direction. The size of these twin domains has been shown to sensitively influence the apparent birefringence in the THz region^5^. Here, measurements were performed on a 0.5 mm thick [001]-oriented single crystal of LaAlO_3_, with twin domains that were small in comparison to the THz spot size. The in-plane crystallographic directions were oriented arbitrarily with respect to the incident THz polarization.

The variation of the change in ellipticity Δ*χ* = *χ*_sample_ − *χ*_reference_ with *ψ*_in_ of pulses after transmission through LaAlO_3_ is presented in Fig. 3(d), at 1.0 (red), 1.5 (green) and 1.75 THz (blue). By considering Δ*χ* rather than *χ* the influence of the finite ellipticity of the reference pulses is removed, and only the influence of the sample on the ellipticity remains. This allows greater sensitivity when investigating materials in which the birefringence is small, as it is at low frequency in this particular sample of LaAlO_3_. Δ*χ* was fit to a cosine model (solid lines), *A*cos(*B*(*ψ*_em_ + *ϕ*)) + *C*, where *A*, *B* and *C* are constants and *ϕ* is a phase offset, which gave Δ*χ* = 0° at −16.5° and 76.0°, corresponding to the directions of the polarization eigenvectors. The directions of the eigenvectors measured here are given to a precision of ±1.25°, limited by the angular step size of the scan.

The change in ellipticity when *ψ*_in_ is parallel to and midway between the polarization eigenvectors is reported in Fig. 3(e). When probing along the eigenvectors Δ*χ* = 0 (green and purple lines), whilst when probing at the midpoints Δ*χ* increases nonlinearly with frequency (red and blue lines) towards the lowest phonon modes of LaAlO_3_^5^. The birefringence of LaAlO_3_ was calculated similarly to the method used previously for ZnO, by assuming a frequency dependent model Δ*n* = Δ*n*_0_ + *βf* ^2^ and calculating the frequency dependent change in ellipticity for certain *ψ*_in_, giving Δ*n*_0_ = 0.0045 and *β* = 0.0065 THz^−3^. As with ZnO, a linear term to Δ*n* was considered, but was found to be negligible for LaAlO_3_. The difference in the frequency dependence to the birefringence in LaAlO_3_ compared to ZnO is due to its lower frequency infrared active phonon modes, at 5.0 and 5.5 THz for the *A*_1_ and *E*_1_ modes respectively^5^, which occur closer to the experimental frequency range.

### RP-THz-TDS of electromagnon absorption in CuO

To demonstrate how RP-THz-TDS can be used to investigate the absorptive properties of anisotropic media, the dependence of the electromagnon absorption on the incident THz orientation angle was investigated in CuO. With a monoclinic crystal structure (space group *C*2/*c*), CuO is a biaxial crystal. CuO exhibits four magnetic phases in zero external field, a high temperature paramagnetic state (PM), a low temperature antiferromagnetic state (AF1), a multiferroic state (AF2) between 213–229.3 K, and an intermediate antiferromagnetic phase (AF3) between 229.3–230 K^34,35^. The multiferroic state hosts a collective excitation known as an electromagnon, an electric-dipole-active magnon, at THz frequencies^7,36^. The electromagnon absorbs light only for electric field oriented close to the [101] direction. Since a cryostat is required to access the temparature range at which the electromagnon is active, rotating the THz polarization state (rather than the sample) is a highly convenient method to probe its anisotropic optical properties. Here, measurements were performed on a 1.3 mm thick single crystal of CuO that was aligned by Laue X-ray diffraction to have a $\mathrm{(10}\bar{1})$ surface normal. Thus, the [101] and [010] directions are in-plane, and the sample was oriented in the spectrometer such that the [101] direction is close to *ψ*_in_ = 0°. RP-THz-TDS was performed at 210 K, in the AF1 phase, and at 215 K, in the AF2 phase.

To examine the electromagnon’s absorption and to precisely determine its selection rule, we examined the temperature-dependent change in absorption coefficient between the AF1 and AF2 phase, expressed as ${\rm{\Delta }}\alpha (\omega ,{\psi }_{{\rm{in}}})=(-\mathrm{2/}d)\mathrm{ln}|\tilde{T}(\omega ,{\psi }_{{\rm{in}}})|$, where *d* is the sample thickness and $|\tilde{T}(\omega ,{\psi }_{{\rm{in}}})|$ is the absolute part of the complex transmission function. To find the transmission function $|\tilde{T}|$ at each value of *ψ*_in_ we found the ratio of the total transmitted intensity using $|\tilde{T}(\omega )|={({|{\tilde{E}}_{{\rm{H}}}^{{\rm{s}}}(\omega )|}^{2}+{|{\tilde{E}}_{{\rm{V}}}^{{\rm{s}}}(\omega )|}^{2})}^{\mathrm{1/2}}/{({|{\tilde{E}}_{x}^{{\rm{r}}}(\omega )|}^{2}+{|{\tilde{E}}_{y}^{{\rm{r}}}(\omega )|}^{2})}^{\mathrm{1/2}}$, where the superscripts s and r denote the sample (at 215 K) and reference (at 210 K) spectra, and the subscripts *x* and *y* denote the horizontally and vertically polarized components, respectively.

**Figure 4:**
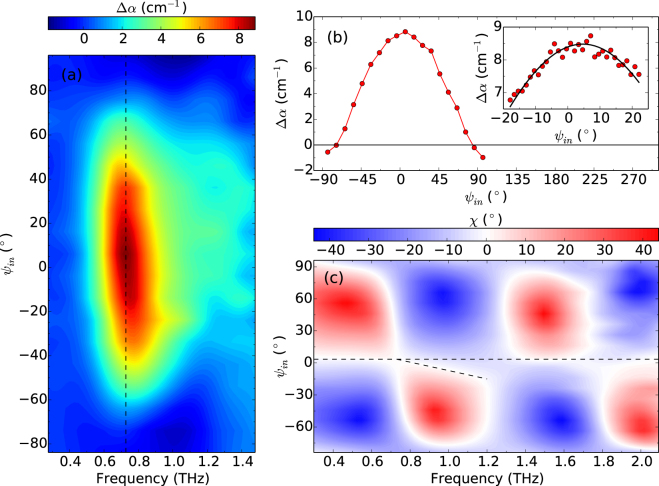
(**a**) Temperature-induced change in terahertz absorption coefficient, Δ*α*, versus incident orientation angle *ψ*_in_ and frequency. The dashed line represents the maximum absorption at 0.72 THz. (**b**) Evolution of Δ*α* with incident orientation angle at 0.72 THz. The inset shows the fit (solid line) and experimental data (red dots) for a scan over the peak of the absorption with a smaller angular step size. (**c**) Ellipticity of pulses after transmission through CuO for various incident orientation angles. The long dashed line at *ψ*_in_ = 4° represents the approximate orientation of the [101] direction. The short dashed line is a guide to the eye highlighting chromatic dispersion.

The change in absorption induced by multiferroicity, Δ*α*(*ω*), as *ψ*_in_ is varied is reported in Fig. 4(a). The electromagnon is evident as a peak in Δ*α* around 0.7 THz with a weaker tail at higher frequencies, and a strength that decreases as *ψ*_in_ moves away from [101] (close to *ψ*_in_ = 0°) towards [010] (close to *ψ*_in_ = ±90°). This verifies that the electromagnon is only excited when applying a THz electric field along the [101] direction, as reported by Jones *et al*.^7^ using measurements at only a few fixed angles. A cut through the peak of the absorption at 0.72 THz is shown in Fig. 4(b), represented by the dashed line in Fig. 4(a). The maximum change in absorption occurs when *ψ*_in_ = 6°, with a precision of 5° defined by half the angular step size of the scan. To more precisely determine the orientation of maximum absorption, an angular range of 40° containing the peak of the absorption was scanned with a smaller angular step size, shown in the inset of Fig. 4(b). The experimental data (dots) were fit (solid line) to a cosine model, *A* cos(2(*ψ*_in_ + *ϕ*)) + *C*, where *A* and *C* are constants and *ϕ* is a phase offset. From this fit the angle of maximum absorption occurs at *ψ*_in_ = 5°. Also in Fig. 4(a) as *ψ*_in_ approaches ±90°, where it is close to the [010] direction, Δ*α* is observed to be negative, corresponding to more transmission in the AF2 phase. This occurs due to the static polarization along [010] in the multiferroic phase altering the absorption of the higher-lying phonon modes^22^.

Due to its monoclinic crystal structure, the dielectric tensor in CuO is

3 $${\boldsymbol{\varepsilon }}=(\begin{array}{ccc}{\varepsilon }_{xx} & 0 & {\varepsilon }_{xz}\\ 0 & {\varepsilon }_{yy} & 0\\ {\varepsilon }_{zx} & 0 & {\varepsilon }_{zz}\end{array}),$$

where $x\parallel a,y\parallel b\,{\rm{a}}{\rm{n}}{\rm{d}}\,c\parallel {c}^{\ast }$ ^37^. The ellipticity after transmission through CuO at 215 K is shown in Fig. 4(c). The region where *χ* ≈ 0 occupies a range of around 6° centered on *ψ*_in_ = 4°, represented by the dashed line in Fig. 4(c). This is in agreement with the angle of maximum Δ*α* determined from the inset of Fig. 4(b), suggesting that this polarization eigenvector coincides with the [101] direction. The birefringence in CuO was calculated using the same method as above for ZnO and LaAlO_3_, and was found to be linearly dependent on frequency, Δ*n* = Δ*n*_0_ + *αf* with Δ*n*_0_ = 0.14 and *α* = 0.03 THz^−1^.

From Fig. 4(c) it can be observed that the eigenvector along [101] demonstrates chromatic dispersion, *i.e.* a frequency dependence to its direction. This can be seen as a deviation in the *ψ*_in_ for which *χ* = 0° away from the dashed line at *ψ*_in_ = 4°, highlighted by the shorter dashed line. The rotation of the polarization eigenvector away from [101] as a result of chromatic dispersion is almost 20° at 1.2 THz. For comparison, Fig. 3(b) shows that the polarization eigenvectors in ZnO occur at a constant *ψ*_in_. In a monoclinic crystal, a preferential triplet of orthogonal directions is not compatible with the crystalline symmetry^38^. The frequency dependence of the complex components of the dielectric tensor causes the principal axes of the crystal, and hence the propagation eigenvectors ***u***_1,2_, to also vary with frequency. In monoclinic crystals two of the principal axes are colour dispersive, while one principal axis has a fixed direction^38^. From equation 3, since there are no off-diagional components to the dielectric tensor involving *y*, the fixed principal axis will occur along the *b* direction, and the two colour dispersive axes will lie in the *ac*-plane. In this particular case, light propagates along the $\mathrm{(10}\bar{1})$ surface normal, which has been found previously to be a principal axis of the dc dielectric tensor^37^. The observation of birefringence for this ***k*** means that $\mathrm{(10}\bar{1})$ is not an optical axis.

## Discussion

A convenient method to study the optical properties at THz frequencies of anisotropic materials was introduced and validated, meeting the criteria of a minimal change in electric field amplitude and polarization state upon polarization rotation across the entire experimental bandwidth. The precision of the ellipticity angle and orientation angle was better than 0.1° from 0.3–2.5 THz. The rotatable polarization method was applied to study a wire-grid polarizer, which was found to produce elliptically polarized THz pulses when the incident pulse was at an arbitrary angle to the wires. The good precision in *ψ* and *χ* was beneficial for the precise determination of the birefringence and polarization eigenvectors of uniaxial (ZnO, LaAlO_3_) and biaxial (CuO) crystals. In the latter case, the polarization-rotation method was used to find the exact angle of peak electromagnon absorption in the improper ferroelectric phase of CuO, to more precisely study its selection rule. This new approach is beneficial for the study of anisotropic compounds, as there is no need to rotate the sample, which can be impractical, for instance for samples held in a cryostat.

## Methods

Interdigitated photoconductive emitters were realized on 0.5 mm-thick semi-insulating GaAs substrates by standard photolithography. One such emitter is shown schematically in Fig. 1(a). The photoactive area was 2.0 mm × 1.3 mm and consisted of gold strips with a 5 *μ*m width and a 5 *μ*m spacing (200 strips within the 2.0 mm width). To avoid destructive interference, further gold strips covered every other gap (Au mask). In order to prevent short circuiting, a 110 nm layer of insulating Al_2_O_3_ vertically separated the mask and bias contacts. Such a thickness was chosen in order to maximize the transmission of the 800 nm photoexcitation beam. The THz generation beam [red waveform in Fig. 1(a)] had a power of 200 mW, and photoexcited carriers in the GaAs substrate. These were accelerated under the applied voltage (±10 V), and generated a THz pulse (blue waveform).

### Data Availability

Data related to this publication is available from the University of Warwick data archive at http://wrap.warwick.ac.uk/89269.
